# Multicomponent stretching and rubber band strengthening exercises do not reduce overuse shoulder injuries: a cluster randomised controlled trial with 579 handball athletes

**DOI:** 10.1136/bmjsem-2021-001270

**Published:** 2022-03-11

**Authors:** Leonard Achenbach, Gunnar Huppertz, Florian Zeman, Johannes Weber, Patrick Luig, Maximilian Rudert, Werner Krutsch

**Affiliations:** 1 Department of Orthopedics, König-Ludwig-Haus, Julius Maximilians University Würzburg, Würzburg, Germany; 2 Department of Trauma Surgery, University Medical Center Regensburg, Regensburg, Germany; 3 Center for Clinical Studies, University Medical Center, Regensburg, Germany; 4 German Handball Federation, Dortmund, Germany

**Keywords:** overuse, intervention, handball

## Abstract

**Objectives:**

Handball is associated with a high risk of overuse shoulder injury. This study investigated if an injury prevention programme effectively reduces overuse injury to the throwing shoulder of handball athletes.

**Methods:**

61 men’s and women’s handball teams (u-19 and senior athletes) were cluster-randomised into an intervention and a control group in the 2019–2020 season. Players of the intervention group regularly carried out an injury prevention programme. Both groups documented overuse shoulder injuries via an online questionnaire every second week. The primary endpoint was the prevalence of overuse injury to the throwing shoulder. Secondary endpoints were the influence of compliance on the primary endpoint and intensity of overuse shoulder symptoms measured by a shortened, handball-specific Western Ontario Shoulder Index (WOSI).

**Results:**

31 teams (295 players) in the intervention group and 30 teams (284 players) in the control group were included for analyses. The overall questionnaire response rate was 61%. The average prevalence of overuse shoulder injury did not significantly differ between the intervention group (n=109, 38.4% (95% CI 32.9% to 44.2%)) and the control group (n=106, 35.9% (95% CI 30.7% to 41.6%), p=0.542). Compliance with the intervention programme did not significantly affect overuse shoulder injury (p=0.893). Using generalised estimating equations for WOSI, the estimated mean for the intervention group was 44.6 points (95% CI 42.0 to 47.1) and 47.6 points for the control group (95% CI 44.9 to 50.3, p=0.111).

**Conclusions:**

A multicomponent exercise programme using rubber bands and stretching did not significantly reduce the prevalence or symptoms of overuse throwing shoulder injury in handball athletes of both sexes. Randomised controlled study; level of evidence I.

**Trial registration number:**

ISRCTN99023492.

**Key messages**

**What is already known on this topic**

- The prevalence of overuse injury of the throwing shoulder and throwing elbow in amateur and recreational handball athletes is unknown.
- No evidence exists regarding the potential benefits of injury prevention programmes for reducing overuse injury to the throwing shoulder for the high-risk group of amateur and recreational handball athletes.

**What this study adds**

- The prevalence of overuse injury (36%) and substantial overuse injury (26%) of the throwing shoulder in adult and U-19 handball athletes is high for both sexes.
- The prevalence of overuse injury (8%) and substantial overuse injury (6%) of the throwing elbow is moderate.
- A multicomponent exercise programme using rubber bands, stretching and partner exercises to improve the glenohumeral range of motion, scapular muscle strength and glenohumeral external rotation strength, did not significantly reduce prevalence or symptoms of overuse throwing shoulder injury in handball athletes.

**How this study might affect research, practice or policy**

- Future exercise programmes should use a more complex injury reduction model and include a higher training stimulus to decrease previously established risk factors, such as external rotation strength.
- Improved understanding of the exact pathomechanism and the factors that may increase the risk of injury in this high-risk group is needed in the future to establish a more effective means to reduce overuse injury.

## Introduction

Handball, one of the most popular team sports globally, is characterised by passing and throwing movements. Throwing the ball and scoring a point is the most important part of the game for field players. A prevalence of 26%–28% of overuse injuries to the throwing shoulder has been described in professional adult handball players of both sexes.^1 2^ In elite youth handball, shoulder injury represents the most frequent overuse injury, with 25%–60% of all injuries.^3–5^ Initial benign overuse injury may manifest into severe pathologies of the throwing shoulder, such as SLAP lesions that must be addressed operatively.^2^ After surgery, low return-to-competition rates to the preoperative level have been described.^6^ Thus, the prevention of shoulder overuse injury is of utmost importance.

To date, little evidence exists regarding the potential benefits of neuromuscular exercise programmes for reducing the prevalence and symptoms of overuse injury to the throwing shoulder for the high-risk group of handball players.^7^ The purpose of this study was to develop an injury prevention programme with sufficient and practicable exercises to reduce injury rates and to analyse its effect on reducing the prevalence and symptoms of overuse injury to the throwing shoulder of handball athletes. It was hypothesised that frequent glenohumeral stretching and shoulder strengthening exercises decrease the prevalence and symptoms of overuse shoulder injury in male and female handball players compared with a control group.

## Materials and methods

This prospective cluster randomised controlled trial (RCT) included male and female handball teams aged over 16 years. This cluster RCT was registered with the International Standard Randomised Controlled Trial Number registry (ISRCTN ID ISRCTN99023492) and took place from 1 July 2019 to 10 March 2020. This report was prepared according to the Consolidated Standards of Reporting Trials Statement 2010 recommendations with extension for reporting cluster-randomised trials.^8 9^

Through public announcement, social media channels and direct contact with the German Handball Federation and regional handball federations, teams were invited to participate in the study before the 2019–2020 season. The target population were handball teams participating in men’s third national league to the lowest league, the women’s first national league to the lowest league, and the under-19 (U-19) national league to the lowest league. After registration by a team official, participating coaches and athletes were sent detailed instructions about the study design and the planned study protocol. Athlete registration took place from 15 May 2019 to 30 June 2019.

Inclusion criteria were being an active handball player in any of the German-speaking leagues and playing at a senior or U-19 level at the time of registration. Exclusion criteria included not having participated in any official match during the 2019–2020 season, change of teams after registration, or fewer than four complete responses to the questionnaires over the season.

### Randomisation

Block-stratified cluster randomisation was used to randomly allocate the handball teams into an intervention group or a control group in a 1:1 ratio. A cluster was defined as a whole club. Randomisation based on individual players or even teams within the same club was not possible because of joint training sessions. Randomisation took place on 1 July 2019, the day after the closure of registration for study participation. An independent statistician generated a randomisation list using the software SAS V.9.4. The procedure proc plan and each participating team were randomised into one of the treatment groups. Block size was defined as 10. Stratification factors were age (U-19 or adult), sex (male or female) and the league level (professional: 1st–3rd national league; amateur: 4–6th league and recreational: 7th league or lower). Teams of the same club were allocated to the same intervention or control group, which led to small imbalances within the strata. The teams of the intervention group were instructed to carry out the exercise programme and the control group teams continued their usual training modules over the study season.

### Injury prevention programme

**Figure 1:**
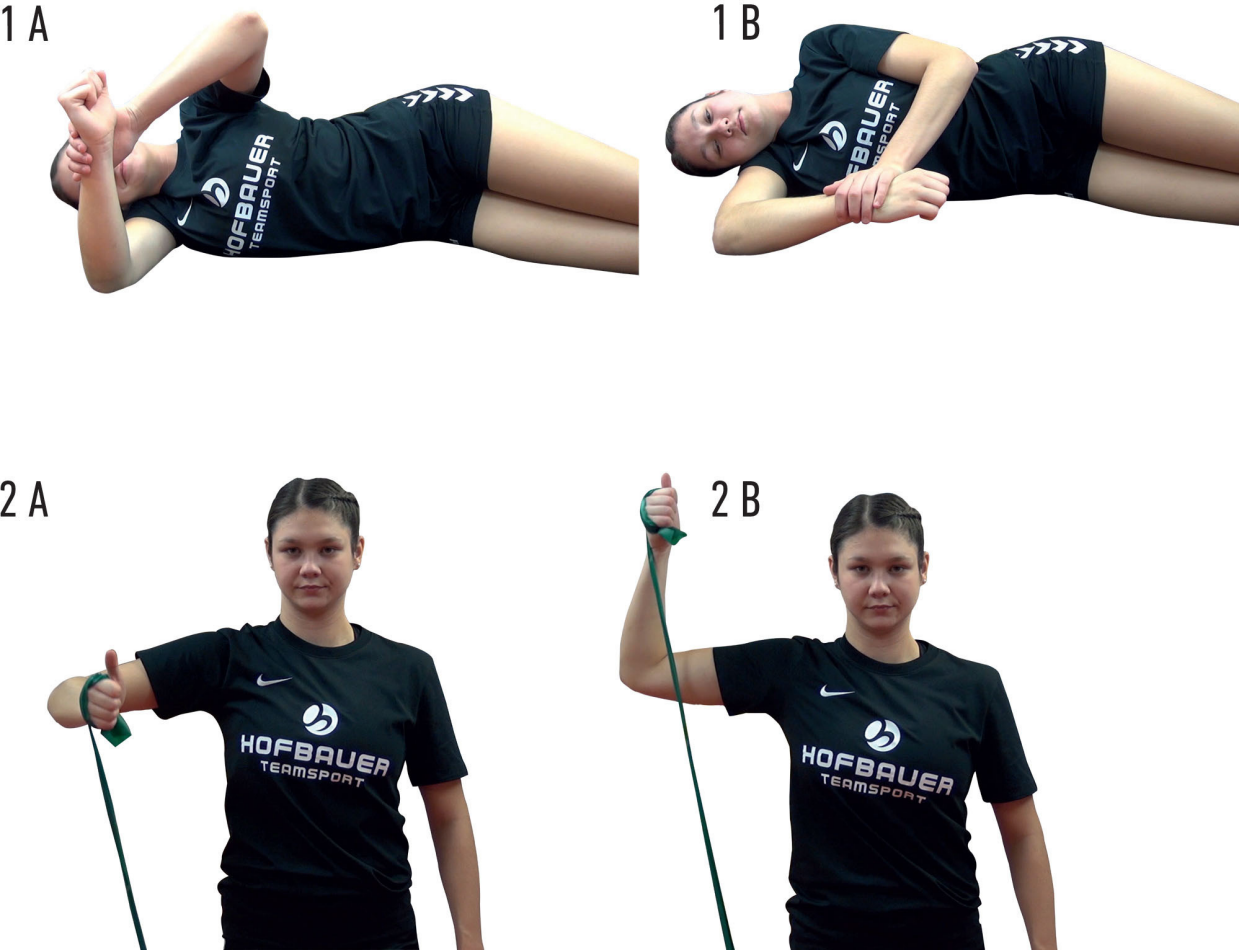
Starting (A) and ending (B) position for (1) glenohumeral stretching exercises and (2) rubber band strengthening exercises.

A handball-specific injury prevention programme was developed for the daily routine in handball by the first author in cooperation with professional coaches of the Bavarian Handball Federation. The exercise programme used in this study was based on identified risk factors for overuse shoulder injuries in throwing sports, especially handball, and on previously established exercise programmes for the shoulder joints.^7 10 11^ The programme blocks included exercises for improving scapular activation, scapular control, scapular strength, glenohumeral external rotation strength and glenohumeral internal range-of-motion (table 1, figure 1, online supplemental appendix).

10.1136/bmjsem-2021-001270.supp1 Supplementary data

**Table 1:** Details of the shoulder exercise programme

Module	Category	Exercise level	Exercise
I	Scapular activation	Beginner	Scapular circles
		Experienced	Scapular circles with bent elbows
		Advanced	Scapular circles with different arm positions
II	External rotation strength	Beginner	Sharapova with rubber bands
		Experienced	External rotation with rubber bands
		Advanced	External rotation partner exercise
III	Scapular strength	Beginner	Reversed snow angel
		Experienced	W, T, Y rubber band exercise
		Advanced	Single-arm W, T, Y rubber band exercise
VI	Scapular control	Beginner	Scapular push-up
		Experienced	Seated wall angel
		Advanced	Y wall slide
V	Rotational internal range of motion		Sleeper’s stretch OR cross-body stretch

The injury prevention programme consisted of 15 min training exercises 2–3 times per week during the preseason lasting 10–12 weeks and 15 min training exercises twice per week during the competition period. During the competition period, one of these two exercises needed to be carried out before a match if the match took place during the week. The programme consisted of five exercise blocks. Coaches were informed that each session should contain at least one exercise from each block. Each block comprised one exercise, which progressed in three steps from easy to more difficult. Exercise progression was decided on individually by the athlete or the team coach. After a player’s progression to the most difficult level of an exercise module, the athletes or team coaches were free to choose between the different exercise levels available.

Ideally, exercises consisted of 2 or 3 sets of 8–10 repetitions to the point of moderate muscle fatigue. Eccentric exercises started with 1 set of 3–4 repetitions to be increased to a maximum of 2 sets of 6–8 repetitions. Static stretches were performed for 40–60 s, comprising 2–3 times 20 s stretches, held at the point of mild discomfort. All exercises could be executed with a rubber band. Partner exercises were included for each block (online supplemental appendix 1).

On the day of randomisation, team coaches and athletes in the intervention group were sent a DVD with detailed information on the injury prevention programme, including instructions on each exercise using pictures and videos, and written information via email about the aims of the injury prevention programme.

### Data assessment

All data management activities were conducted with Research Electronic Data Capture (REDCap), a web-based clinical data management system.^12^ Clinical data were collected and stored on a local server operated by the data processing centre of the University of Regensburg.

The personal and anthropometric data of the players were collected through a standardised baseline questionnaire at the beginning of the season. Players then received an email with the link to an online questionnaire that had to be filled in every second Monday morning throughout the season with the exemption of a 4-week break over the Christmas holidays. The questionnaire consisted of the Oslo Sports Trauma Research Centre (OSTRC) overuse questionnaire and a short newly developed handball-specific Western Ontario Shoulder Index (WOSI).^13 14^

The OSTRC is a set of four questions that can be added to a maximum score of 100 arbitrary units. The validated and translated German version of the OSTRC overuse injury questionnaire^15^ was used. We modified the questionnaire to be able to also assess the last 2 weeks instead of only 1 week as intended in the original questionnaire. Furthermore, compliance with the full exercise programme was self-reported by the athletes and was measured through the number of times the programme had been completely carried out over the past 2 weeks. Finally, the athletes reported their training and match exposure per minute over the past 2 weeks.

### Development of short WOSI

The WOSI is a set of 21 questions relating to shoulder symptoms.^14^ The topics of the questions are related to physical symptoms, sports, recreation, work, lifestyle and emotions. The questions are rated from 0 to 100 arbitrary units.

The number of questions of the WOSI was decreased for the short handball WOSI to lower the question load of amateur athletes while maintaining specificity to overuse symptoms of the throwing shoulder in handball.

The five questions from the WOSI were calculated from an ongoing prospective study on overuse shoulder symptoms in handball that had started in 2016. In that study, youth elite handball players were asked to complete the WOSI five times during one season if they had sustained an overuse injury to their throwing shoulder in the period between the last questionnaire and the day of assessment. At the time of developing the questionnaire for the current randomised trial, the study team had received 91 answers. The answers were further analysed with a predefined sum of over 420 points of a maximum of 2100 points.^4^ The five questions with the highest average (from 0 to 100) were chosen for inclusion in the prevention questionnaire. The five questions used in this study for the short handball WOSI were:

- How much pain do you experience in your shoulder during overhead activities?
- How much weakness or lack of strength do you experience in your shoulder?
- How much clicking, cracking or snapping do you experience in your shoulder?
- How much have the symptoms in your shoulder affected your ability to perform the specific skills required for your sport or work? (If your shoulder affects both sports and work, consider the most affected area).
- How concerned are you about the symptoms in your shoulder becoming worse?

### Primary and secondary endpoint

Overuse injury to the throwing shoulder and elbow was defined as >0 arbitrary units of a maximum of 100 of the OSTRC score and assessed each questionnaire.^13^

The primary endpoint prevalence of overuse of the throwing shoulder was defined as at least one injury recording during the entire season. We calculated the prevalence of shoulder problems in both groups by dividing the number of players who had reported any problem (ie, anything but the minimum value in any of the four questions) by the number of questionnaire respondents.^13^

Secondary endpoints were the prevalence of substantial overuse shoulder injury defined as athletes who selected option three or more in question 2 or question 3 and prevalence and intensity of elbow overuse injury.^13^ 13 Further subanalysis was carried out for the intensity of symptoms, defined as the average of the five questions from the short handball WOSI questionnaire. The compliance was analysed as described in the literature. Performing the complete exercise programme on average weekly less than once was defined as ‘low’, between once and twice as ‘medium’ and more than twice as ‘high’.^7^ The same analysis was performed for overuse elbow injury.

### Sample size calculation

Sample size was calculated based on the primary endpoint of prevalence of overuse injury to the throwing shoulder in handball athletes within one season. The prevalence of 28% was based on the percentage reported in the recent literature.^7^ The study aimed to achieve a reduction by 10%, from 28% to 18%, which corresponds to a relative reduction of 36%. Based on Andersson *et al* who found a mean difference of 6%, we considered a reduction of 10% realistic with our exercise programme in a mixed professional and amateur study population. To achieve this effect with a power of 80%, an estimated intraclass correlation coefficient of 0.05, and an average team size of 14 athletes with an error type 1 of maximum 5%, 33 teams per group and thus a total of 66 teams with n=924 athletes had to be included.

### Statistical analysis

All analyses were performed on the full analysis set, defined by the intention-to-treat (ITT) population. The ITT population was defined as all players who participated in the trial (ie, all players of a randomised team) and had at least three complete questionnaires. No analyses regarding the per-protocol population were considered because almost none of the players had completed all questionnaires.

Patient characteristics are summarised as mean and SD or frequency counts (percentages).

Analysis of the primary and secondary endpoints: The primary endpoint ‘prevalence of overuse injuries of the throwing shoulder’ and the secondary outcome endpoints were compared between the two study groups employing a generalised estimating equation (GEE) model and an exchangeable covariance matrix. OR and corresponding 95% CIs are reported. The significance level was set to p<0.05.

The statistical analysis was done in an unblinded manner by the Centre for Clinical Studies using the software SAS V.9.4 (SAS Institute).

### Patient and public involvement

Patients and/or the public were involved in the design, or conduct, or reporting, or dissemination plans of this research. Refer to the Methods section for further details.

## Results

**Figure 2:**
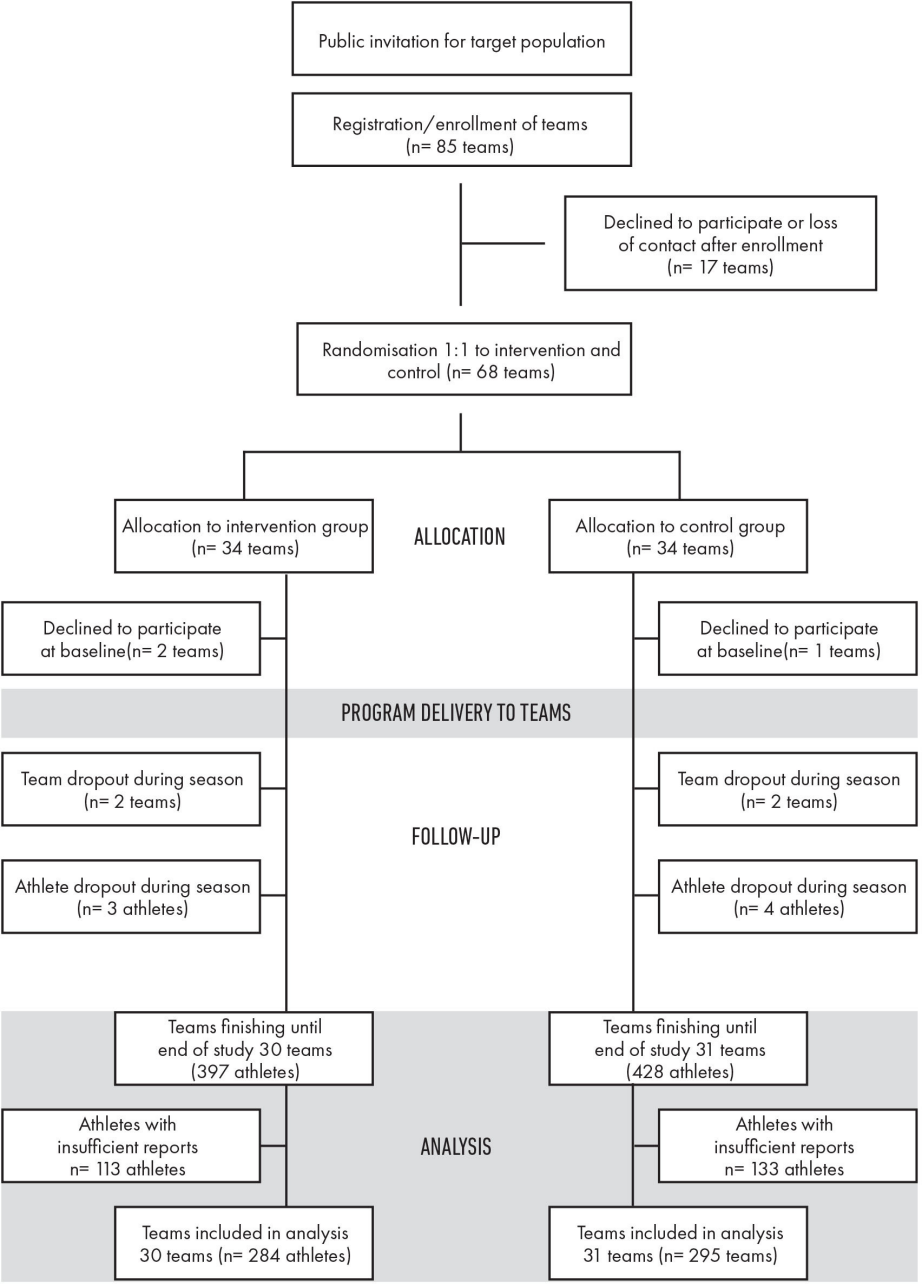
The CONSORT flow diagram. CONSORT, Consolidated Standards of Reporting Trials.

Of the 85 initially registered teams, 24 were excluded due to loss of contact after enrolment or directly after randomisation or the inability to provide the required documentation. Of the 61 teams (n=825) included in the study, 7 players dropped out because they had stopped playing for their team during the study period or because their parents had withdrawn their consent (table 2). Finally, 246 players were excluded due to the lack of the minimum number of completed questionnaire responses (at least three) or because they had stopped playing for their team during the study period or because their parents had withdrawn their consent. This exclusion resulted in n=579 analysable players, with n=284 players (30 teams) in the intervention group and n=295 players (31 teams) in the control group (figure 2). Of 61 teams, 56 clusters with a range of 1–3 teams per club were included. The intervention and control groups showed similar anthropometric data (table 3).

**Table 2:** Distribution of teams according to age, sex and playing level

		Recreational level	Amateur level	Professional level
Intervention group	Men (senior/U-19)	6/0	10/1	0/1
	Women (senior/U-19)	4/0	6/0	2/0
Control group	Men (senior/U-19)	3/1	7/1	0/5
	Women (senior/U-19)	4/1	6/1	1/1

**Table 3:** Anthropometric and handball-specific data

	Intervention group (n=284)		Control group (n=295)	
	Male athletes (n=161)	Female athletes (n=123)	Male athletes (n=159)	Female athletes (n=136)
Height (cm)	185.1±7.3	172.5±6.8	185.4±6.7	169.5±6.4
Weight (kg)	84.3±14.7	70.3±11.0	84.3±11.9	68.2±12.9
Handball experience (years)	14.0±5.4	16.3±5.2	14.3±6.4	13.1±6.2

Mean±SD.

### Response rate

The season was prematurely finished after 17 (81%) instead of the planned 21 questionnaires because of the COVID-19 lockdown in Germany. The response rate had steadily declined over the course of the season, with a response rate of less than 45% for both groups in the last five questionnaires. The overall response rate for the 17 questionnaires during the season was 61%. No difference was seen between the intervention group (60%) and the control group (61%, n.s.).

### Primary and secondary outcome

**Figure 3:**
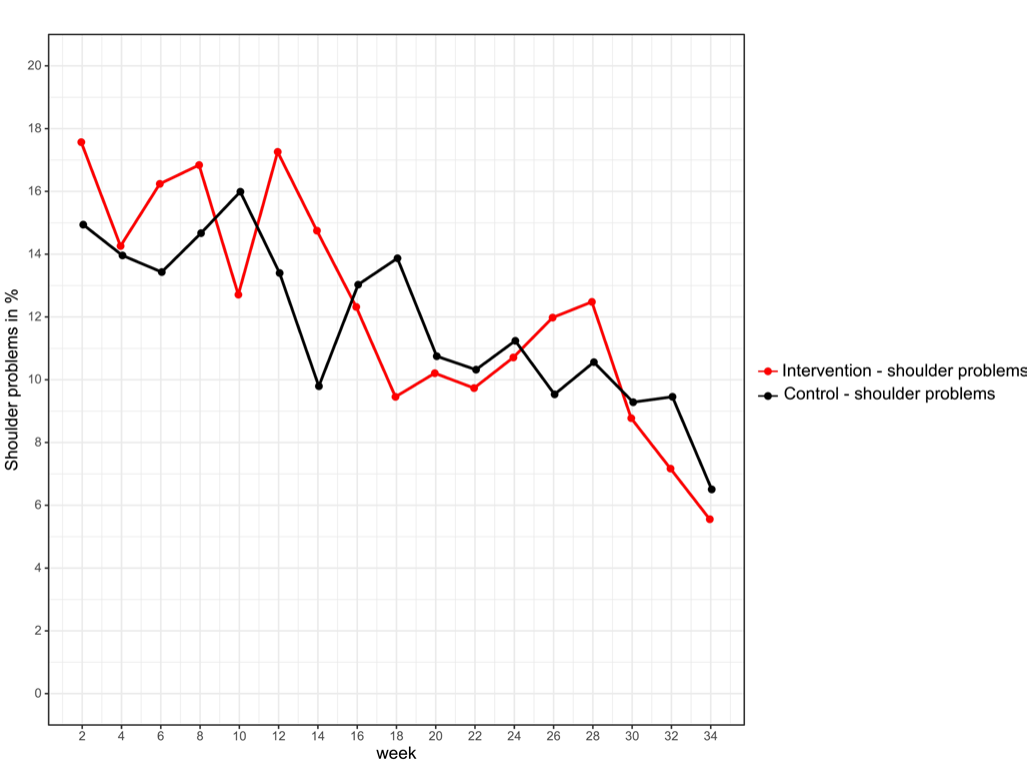
Prevalence of shoulder problems in the intervention (red) and control group (black).

**Figure 4:**
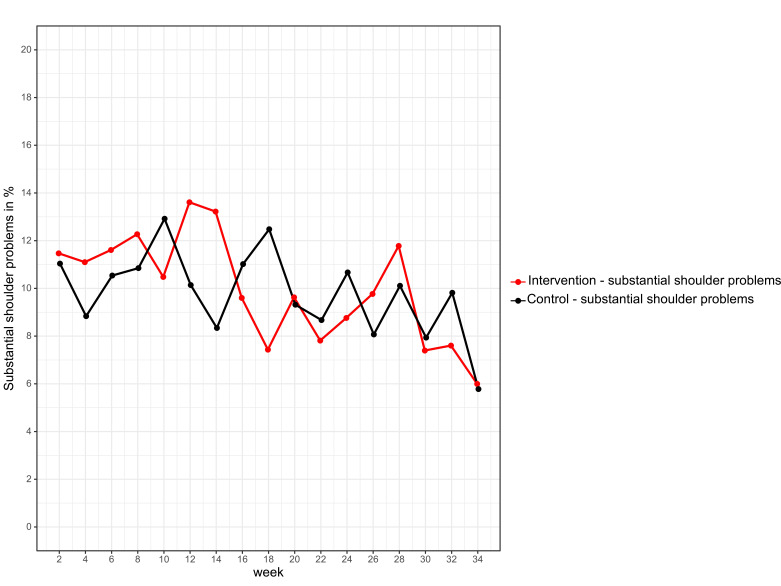
Prevalence of substantial shoulder problems in the intervention (red) and control group (black).

No significant difference in the prevalence of overuse shoulder injury was found between the intervention group (n=109, 38.4% (95% CI 32.9% to 44.2%)) and the control group (n=106, 35.9% (95% CI 30.7% to 41.6%)) with an absolute risk reduction of −2.5% (95% CI −10.3% to 5.4%), p=0.542). No significant difference could be seen for substantial overuse shoulder injury between the intervention group (n=90, 31.7% (95% CI 26.6% to 37.3%)) and the control group (n=78, 26.4% (95% CI 21.7% to 31.8%)) with an absolute risk reduction of −5.3% (95% CI −12.6% to 2.1%), p=0.164). GEE did not yield any significant differences between the two groups for the primary endpoint overuse shoulder injury (p=0.858) and substantial overuse shoulder injury (p=0.739) (figures 3 and 4).

**Figure 5:**
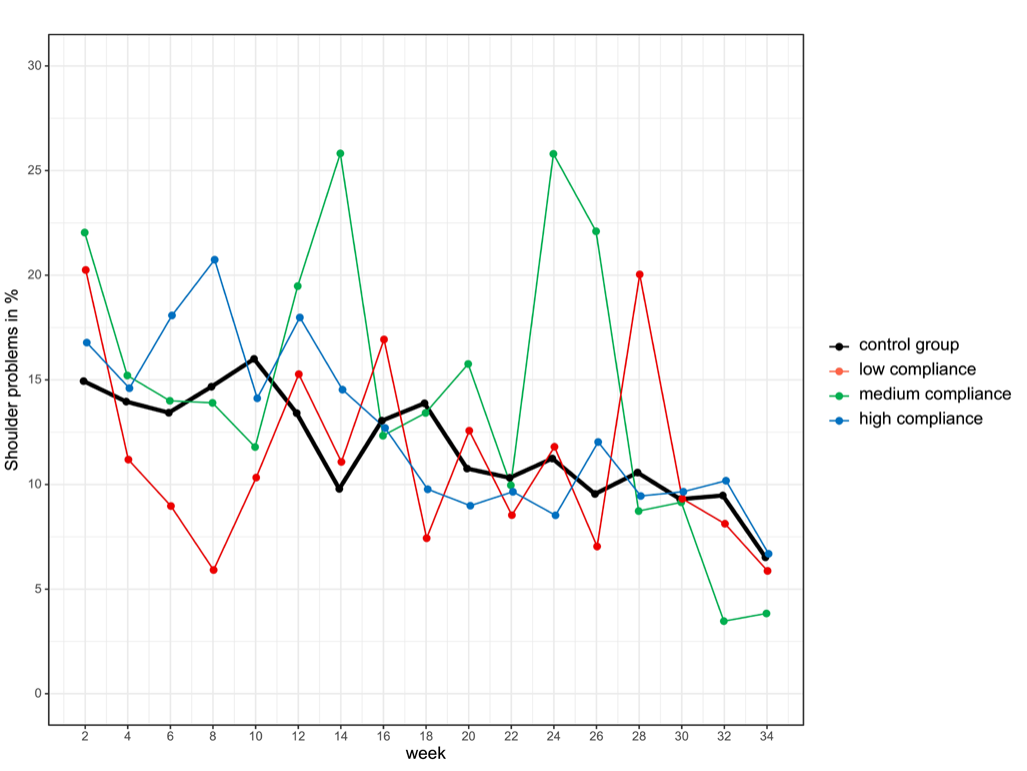
Prevalence of shoulder problems in the intervention group with low (red), medium (green) and high (blue) compliance and control group (black).

**Figure 6:**
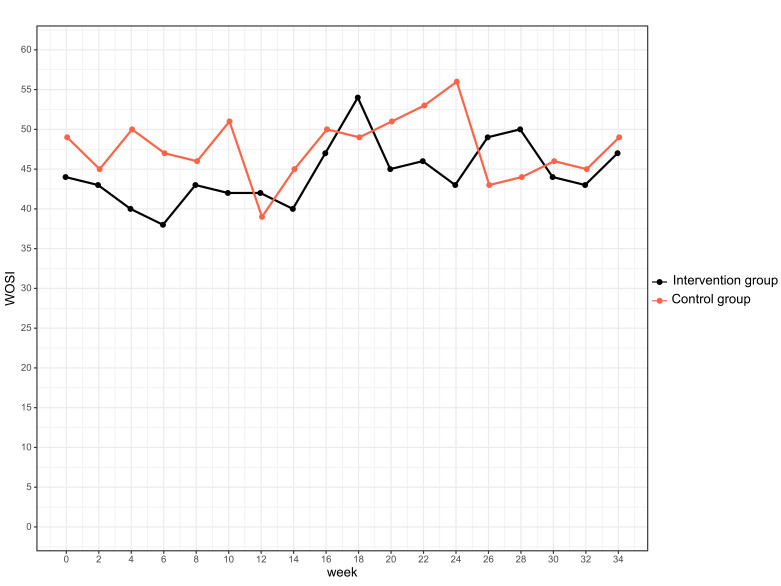
Average of overuse shoulder symptoms in the intervention (red) and control group (black) measured by an average of five handball-specific questions of the Western Ontario Shoulder Index (WOSI) questionnaire.

The intervention group demonstrated very good compliance with the exercise programme: Of 284 athletes, 64% had high compliance, 14% medium compliance and 11% low compliance. Compliance with the intervention programme did not significantly affect overuse shoulder injury (p=0.495) or substantial overuse shoulder injury (p=0.176). GEE was calculated for compliance (figure 5). No differences were found for short handball WOSI scores (figure 6). The estimated means for the intervention group was 44.6 points (95% CI 42.0 to 47.1) and 47.6 points for the control group (95% CI 44.9 to 50.3). The estimated difference was 3.0 points (95% CI 0.7 to 6.8, p=0.111). There were no significant sex differences.

The average prevalence of overuse elbow injury did not significantly differ between the intervention group (n=20, 7.0% (95% CI 4.6% to 10.6%)) and the control group (n=24, 8.1% (95% CI 5.5% to 11.8%), p=0.620) (table 4). The average prevalence of substantial overuse elbow injury did also not significantly differ between the intervention group (n=16, 5.6% (95% CI 3.5% to 9.0%)) and the control group (n=18, 6.1% (95% CI 3.9% to 9.4%), p=0.811) (table 5).

**Table 4:** Prevalence of elbow injury

	Men		Women		All	
	No	Yes	No	Yes	No	Yes
Intervention	148 (92%)	13 (8%)	116 (94%)	7 (6%)	264 (93%)	20 (7%)
Control	147 (92%)	12 (8%)	124 (91%)	12 (9%)	271 (92%)	24 (8%)
P value	0.860		0.334		0.620	

**Table 5:** Prevalence of substantial elbow injury

	Men		Women		All	
	No	Yes	No	Yes	No	Yes
Intervention	150 (93%)	11 (7%)	118 (96%)	5 (4%)	268 (94%)	16 (6%)
Control	151 (95%)	8 (5%)	126 (93%)	10 (7%)	277 (94%)	18 (6%)
P value	0.496		0.258		0.811	

## Discussion

The most important finding of this cluster-randomised controlled study was that the exercise programme did not significantly reduce the prevalence or symptoms of overuse injuries of throwing shoulders in handball athletes. The aim of the study to achieve a reduction in overuse injuries by 10% could not be reached.

The study group comprised primarily amateur and recreational athletes of both sexes. The prevalence of overuse injury (36%) and substantial overuse injury (26%) of the throwing shoulder in the control group of adult and U-19 handball athletes was higher than in previous research.^1–5^ The weekly prevalence steadily declined from a maximum of 15% at the beginning of the season to 7% at the end of the study. We did not find any specific causalities, such as a parallel decline in response rate or any sub-group trends. We also found a high prevalence of overuse elbow injury (8%) and substantial overuse elbow injury (6%). The throwing elbow and especially the throwing shoulder are prone to overuse injury prompting intervention from medical personnel involved in handball.

### Risk factor model

The occurrence of overuse injury of the throwing shoulder is thought to be caused by modifiable risk factors. The exercise programme used in this study aimed to reduce previously proposed risk factors for overuse shoulder injuries in elite throwing sports, especially handball, that is, reduced external rotation strength, scapular dyskinesia and glenohumeral internal rotation deficit, thereby supporting the capacity of the throwing shoulder to better meet the demands of handball. The programme was based on previously established exercise programmes for the shoulder joints.^7 12^ The programme was implemented at the start of the preseason, allowing sufficient time to develop a training effect before the beginning of the competitive season matches.

However, this risk factor model was proposed for elite athletes. Yet most of the teams participating in this study played at the amateur and recreational level, which may have different underlying pathomechanism for shoulder overuse injuries than those identified in elite sports. Using this risk factor model for mainly amateur and recreational athletes may have been too simplified and future exercise programmes should use a more complex injury reduction model.

### Reduction of proposed risk factors

The proposed risk factors may need reconsideration because the exercise programme did not help reduce the prevalence of overuse shoulder injury.

Because scapular dyskinesia had been proposed to be an associated factor for the development of overuse injury,^4 16 17^ our programme included exercises for improving scapular neuromuscular control and periscapular muscle strength. However, scapular dyskinesia may be secondary to another underlying pathology, as identified in a recent review.^18^

A further proposed risk factor is decreased external rotation strength.^4 16 19 20^ Here, the strength of 75% or less compared with the internal rotation strength of the same shoulder may be clinically relevant.^4 19^ The exercises used in this study were similar to the programme used by previous research and to a recent DELPHI consensus statement.^7 21^ However, to date, research about specific training programmes to improve external rotation strength is inconclusive about their effectiveness.^22 23^ Because the exercises used in this study were very similar to that research, this similarity may explain the ineffectiveness of the programme in our study. Thus, future exercise programmes may include a higher training stimulus than rubber bands and partner exercises to gain external rotation strength.

The addition of exercises to stretch the glenohumeral joint’s posterior capsule and subsequently improve the throwing shoulder’s internal rotation has been described with positive effects.^24^ However, it is inconclusive whether glenohumeral internal rotation deficits contribute to the development of overuse injuries in handball.^2 4 16 25–29^

### Comparison to previous research

This result confirms the ineffective preventive potential of exercises used in previous research of overuse injuries in recreational tennis players.^30^ Still, it is in contrast to other exercise programmes that reduced overuse injuries in the throwing shoulder in Norwegian elite handball athletes by 28%^7^ or in elite youth baseball athletes with a HR of 1.940 (95% CI 1.175 to 3.205).^31^ However, it is interesting to note that both studies confirming the effect of exercise programmes do not achieve the compelling results of other injury prevention programmes, such as reduction of acute severe non-contact knee injuries by 51%,^32^ which has also been validated in handball,^33–35^ or of other overuse injuries, such as a 41% reduction in adductor muscle overuse injury in football.^36^

The exercises used in this study differed only slightly from the effective exercise programmes that also aimed to improve glenohumeral range of motion, scapular muscle strength and glenohumeral external rotation.^7 31^ However, the cited two studies included further exercises for improving thoracic mobility, the kinetic chain, single-leg stability and a set of multiple stretching exercises for the upper extremities. These differences may be the reason for their positive effect. Here, further research is necessary.

Another difference was the implementation method: for both programmes, coaches and athletes had been instructed in the proper performance of the exercises, whereas in our study, coaches and athletes had not been supervised, which may have impaired the quality of the exercises.^30^ However, this non-supervised approach has been previously used effectively in RCTs in handball to reduce injuries to the lower extremities.^33^ In addition, the effects of the exercise programme were not evaluated, and thus, the direct effect of the exercises could not be obtained.

The results of this study show that the reduction in overuse injuries to the throwing shoulder is, to date, not yet sufficiently understood. Thus, an improved understanding of the exact pathomechanism and the factors that may increase the risk of injury in this high-risk group are needed in the future. One important factor that increases injury risk in addition to neuromuscular risk factors is the training load^19 37^ which has not been addressed in our study.

### Strengths and limitations

The strength of this intervention study was its prospective randomised controlled design with a high number of clusters and participants, its questionnaires every 2 weeks, and the compliance with the exercise programme to counteract previously identified risk factors specific to the throwing shoulder of handball athletes. The questionnaires and the high number of 17 questionnaires ensured a high response quality for prevalence and symptoms of overuse symptoms. This study has some limitations, such as the premature ending of the season due to the COVID-19 pandemic, a moderate response rate in the last weeks before the end of the season, and a moderate drop-out rate, especially after randomization. The initially calculated sample size could not be reached with 61 teams and 579 athletes included in the final analysis. Thus, the study was underpowered in terms of the initial assumption of the effect of the training programme. The registration trial protocol was retrospectively published online due to technical problems. The final report deviated from the trial protocol by the premature ending and correcting the labelling of the parameter intensity of overuse injury as a secondary endpoint. However, none of the endpoints reached any significance. Although the reporting of overuse injury by self-report questionnaires is feasible for a large cohort, it may not be sufficient for determining the specific diagnosis of individual athletes. Furthermore, the high number of questionnaires may have resulted in a disproportionate questionnaire load, which may be—at least in part—responsible for a decreasing response rate throughout the season for primarily amateur and recreational athletes.

## Conclusion

A multicomponent exercise programme using rubber band, stretching and partner exercises did not significantly reduce prevalence or symptoms of overuse injury to the throwing shoulder in primarily amateur and recreational handball athletes of both sexes.

## Data Availability

Data are available on reasonable request.
