# Supplementary material for: Multicomponent stretching and rubber band strengthening exercises do not reduce overuse shoulder injuries: a cluster randomised controlled trial with 579 handball athletes
Source: BMJ Open Sport Exerc Med. 2022 Mar 11;8(1):e001270. doi: 10.1136/bmjsem-2021-001270 (PMC8919472; doi:10.1136/bmjsem-2021-001270)
Supplement: Supplementary data [file bmjsem-2021-001270supp001.pdf]

SHOULDER ABC PREVENTION EXERCISES

This programme consists of five exercise blocks. Each session should contain at least one exercise from each block. The programme should be completed before every handball match and at least once per week in training sessions. In weeks without any matches, the programme should be completed at least twice. The order of exercises should be maintained, and the **Shoulder ABC** should be used before carrying out any passing or throwing series. The shoulder range-of-motion exercises should be done after training sessions or matches.

The level of workout can be increased by switching from the beginner's exercises to experienced exercises and advanced exercises. The number recommended of repetitions are 2-3 sets of 8-10 repetitions or 2-3 sets of 20 seconds. At the beginning of the (eccentric) partner exercises of shoulder strength, 1 set of 3-4 repetitions is recommended, which may then be increased to 2 sets of 6-8 repetitions. Single arm exercises may be carried out just with the throwing arm.

Achenbach L, *et al. BMJ Open Sp Ex Med* 2022; 8:e001270. doi: 10.1136/bmjsem-2021-001270

SHOULDER ABC EXERCISE BLOCKS

Warm Up

1 Scapular activation

2 Shoulder strength

3 Scapular strength

4 Scapular control

5 Shoulder range-of-motion (after matches and training sessions)

Achenbach L, *et al. BMJ Open Sp Ex Med* 2022; 8:e001270. doi: 10.1136/bmjsem-2021-001270

SCAPULAR ACTIVATION

BEGINNER

Scapular circles

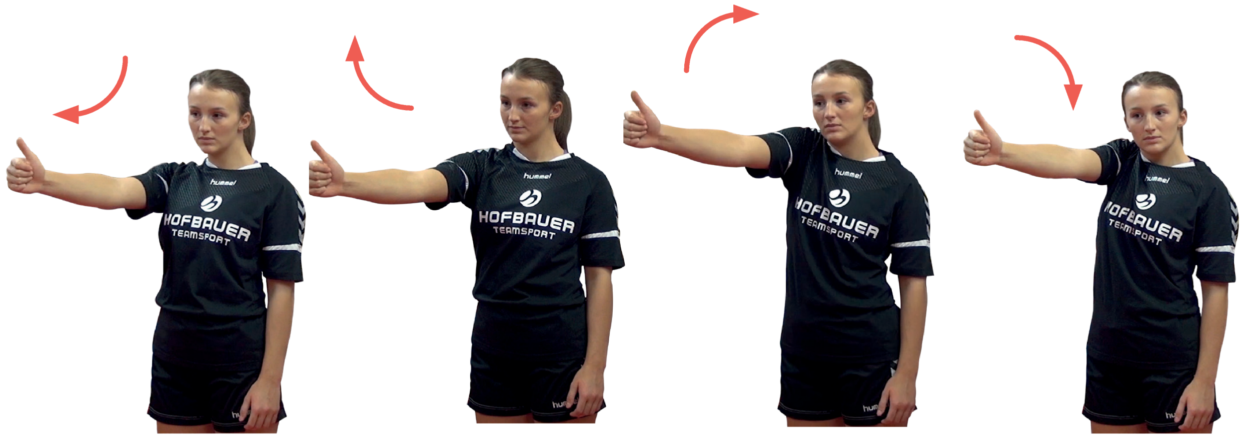

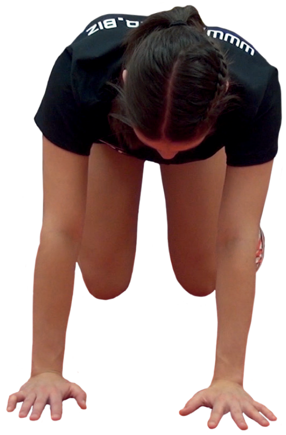

Move your shoulder blades up, forward, down and back. Increase the flow of the circular movement.

- ALTERNATIVE EXERCISE: SHOULDER STAND
  Lock your shoulder girdle and lift your knees from the ground.

SCAPULAR ACTIVATION

EXPERIENCED

Scapular circles with bent elbows

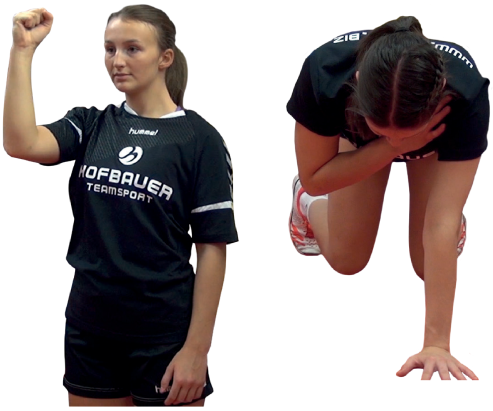

Modify the position of your elbows from straight to bent.

- ALTERNATIVE EXERCISE: SHOULDER TAP
  Unlock one hand and touch your opposite shoulder.

ADVANCED

Scapular circles with different arm positions

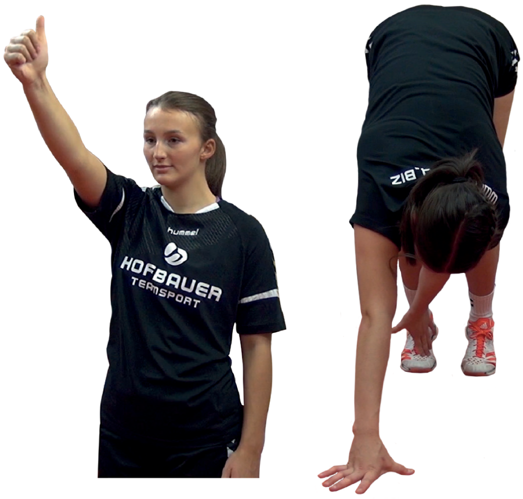

Modify the direction of your shoulder movement.

- ALTERNATIVE EXERCISE: SINGLE ARM PYRAMID
  Build a human pyramid and touch one hand
  with your opposite hand.

SHOULDER STRENGTH

BEGINNER

Sharapova with rubber bands

Imitate climbing a ladder up and down with alternating arm movement on a wall. Keep tension in the rubber bands during the entire exercise.

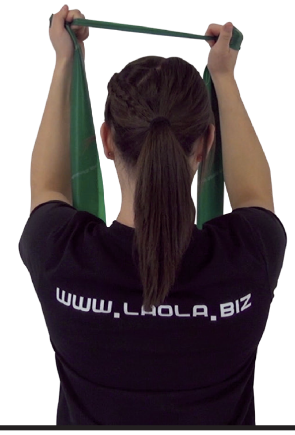

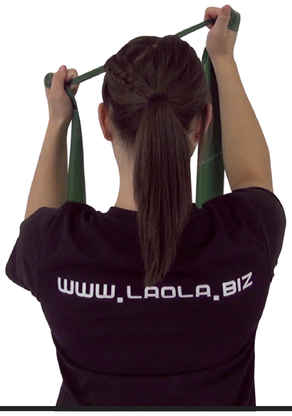

SHOULDER STRENGTH

EXPERIENCED

External rotation with a rubber bands at 90°

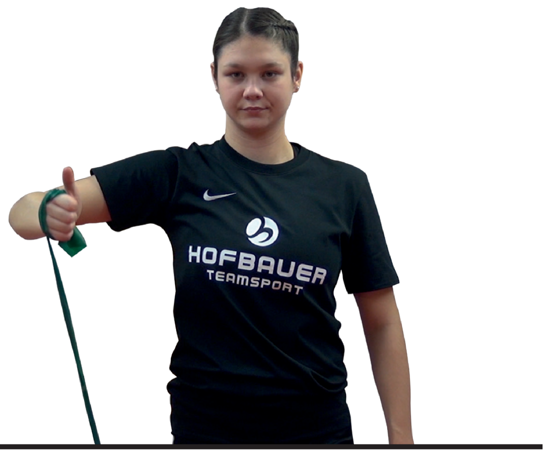

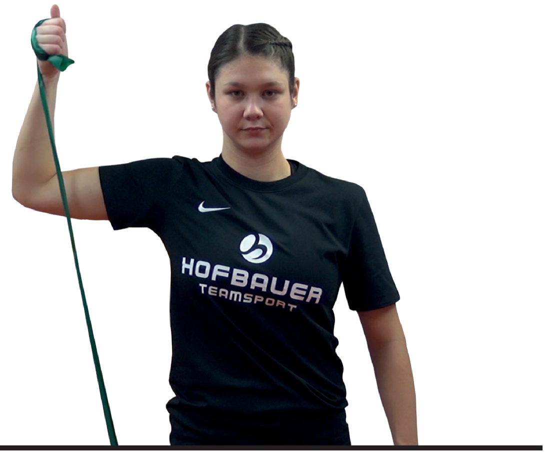

Keep tension in the rubber band at the starting position with the forearm parallel to the ground. Rotate backwards and upwards and get slowly back to the starting position. Keep your elbow at shoulder height during the entire exercise.

SHOULDER STRENGTH

ADVANCED

External rotation partner exercise

Resist but allow partner to push your arm forwards and towards the ground. Keep your elbow at shoulder height during the entire exercise.

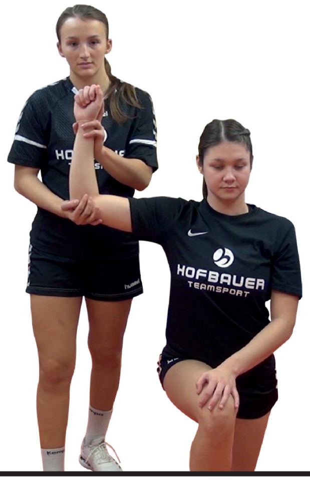

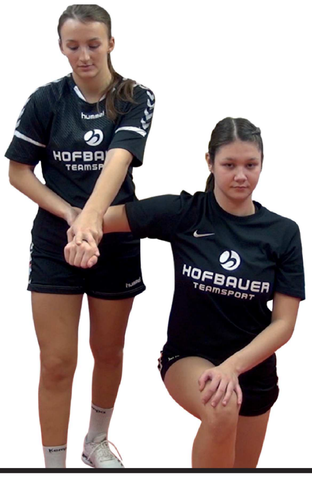

Achenbach L, *et al. BMJ Open Sp Ex Med* 2022; 8:e001270. doi: 10.1136/bmjsem-2021-001270

SCAPULAR STRENGTH

BEGINNER

Reversed snow angel

In prone position, move the handball from one hand to the other over your head and behind your back.

- ALTERNATIVE EXERCISE: WITH OBSTACLES
  Increase difficulty with obstacles placed in the path of the arms.

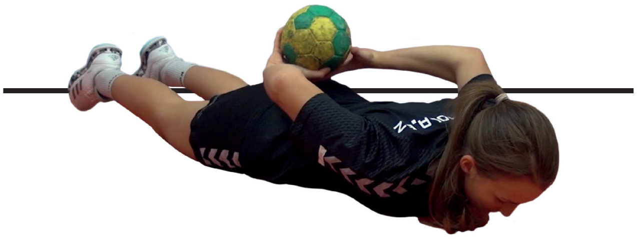

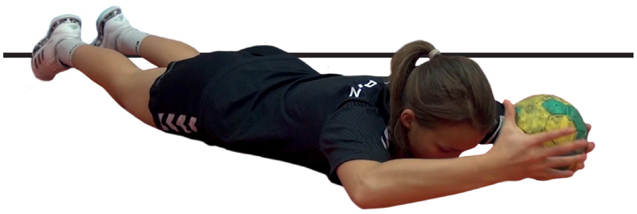

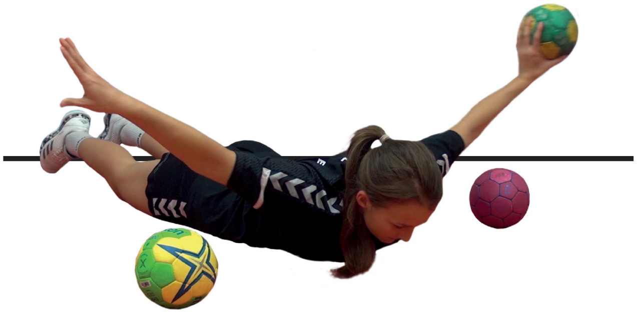

SCAPULAR STRENGTH

Aim backwards with thumbs

EXPERIENCED

W, T and Y rubber band exercise

Keep tension in the rubber band at the starting position.
Pull rubber band with straight arms from starting to end position.
Reach W, T or Y position. Alternate end positions.

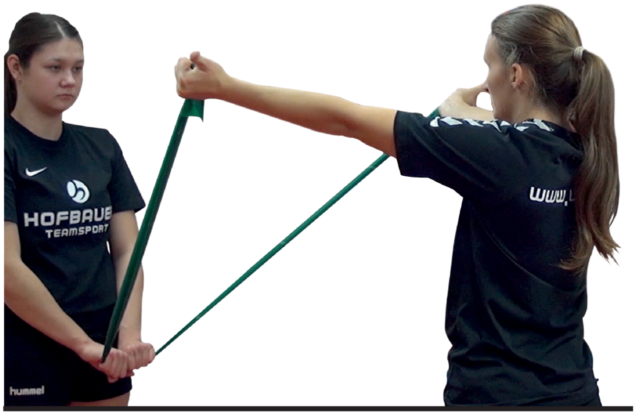

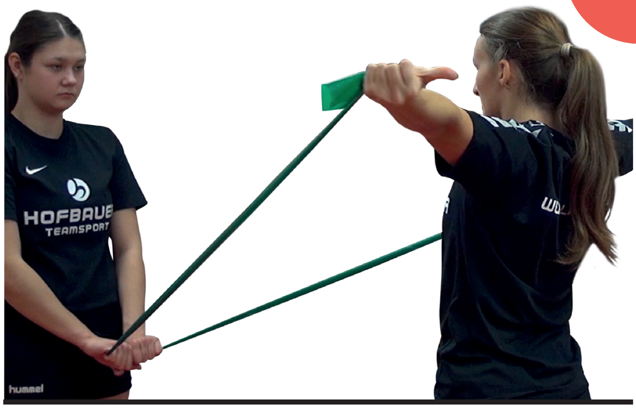

T

Achenbach L, *et al. BMJ Open Sp Ex Med* 2022; 8:e001270. doi: 10.1136/bmjsem-2021-001270

SCAPULAR STRENGTH

ADVANCED

Single arm W, T and Y rubber band exercise

Reach W, T or Y position. Pull your opposite (non-throwing) shoulder in opposite direction or lock rubber band with your foot.

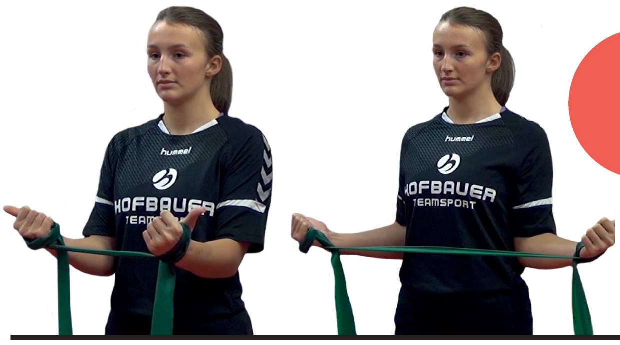

W

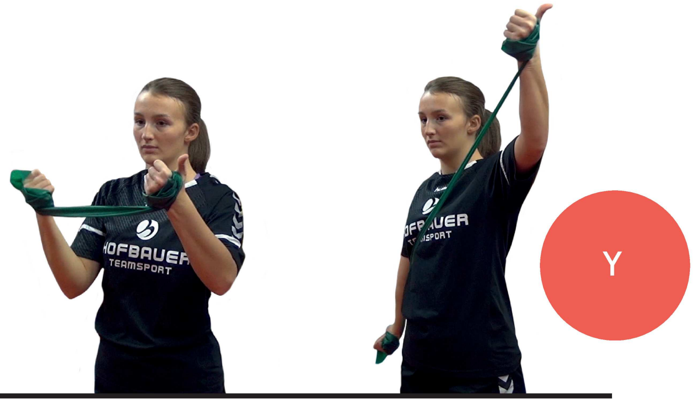

Y

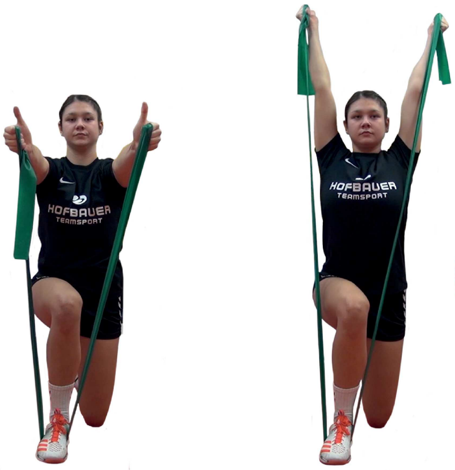

Y

SCAPULAR CONTROL

BEGINNER

Scapular push-up

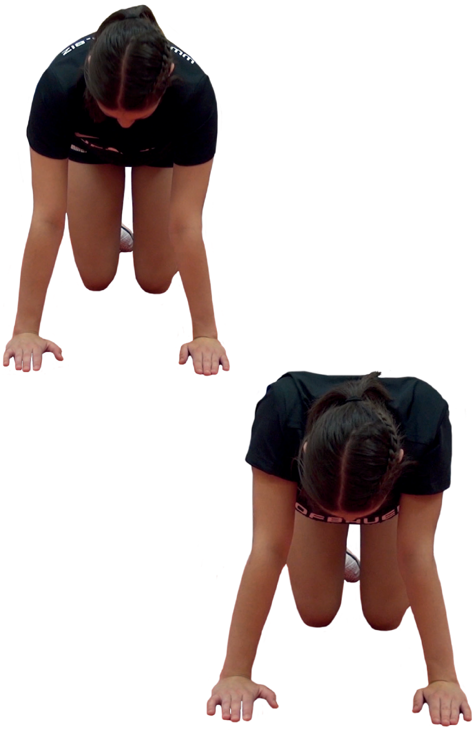

Adopt a push-up position with straight elbows, pinch your shoulder blades together and push them forward.

- ALTERNATIVE EXERCISE: WITH KNEE LIFT OFF
  Increase difficulty by lifting your knees from the ground.

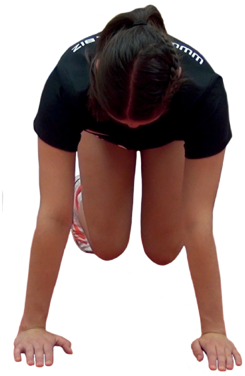

SCAPULAR CONTROL

EXPERIENCED

Seated wall angel

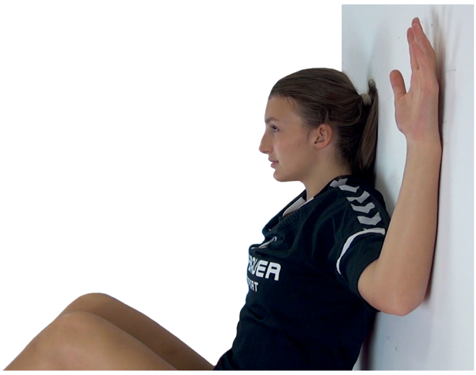

Adopt a sitting position with your knees pulled to the chest, move your elbows up and down along the wall. Keep contact to the wall with your forearm during the entire exercise.

• ALTERNATIVE EXERCISE: WITH RUBBER BAND

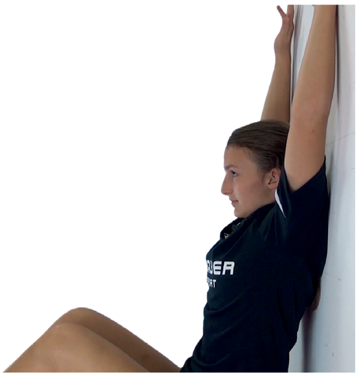

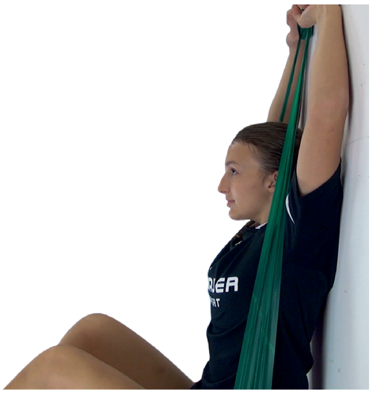

SCAPULAR CONTROL

ADVANCED

Y wall slide

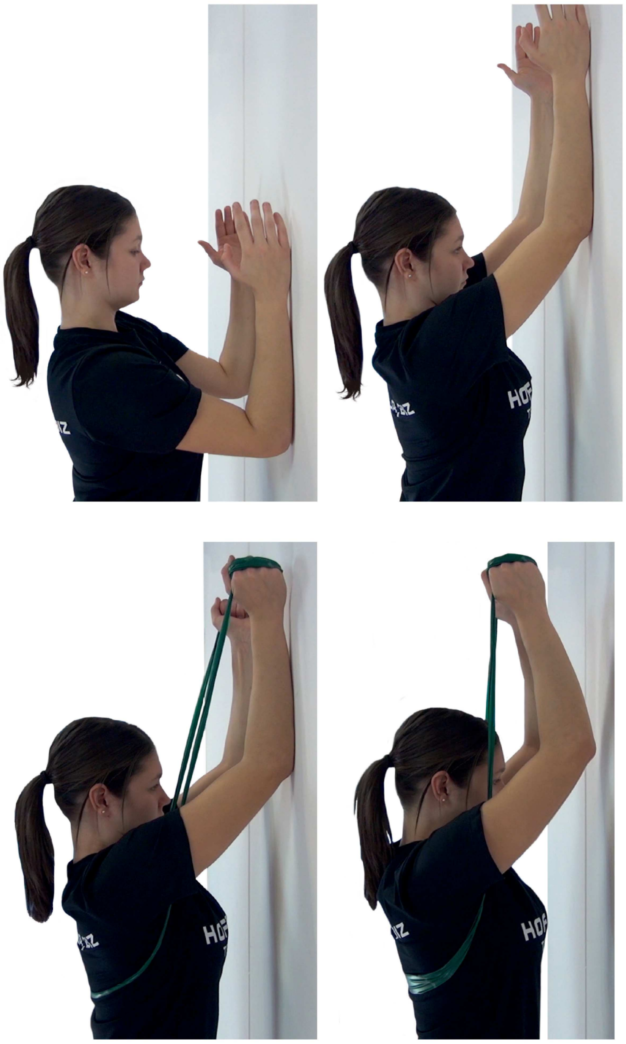

Adopt a standing position with the entire length of your forearms touching the wall, slide upwards without losing contact to the wall. Increase difficulty by lifting forearms to the (highest) end position.

- ALTERNATIVE EXERCISE: WITH RUBBER BAND

- ALTERNATIVE EXERCISE: WITH FOREARM LIFT OFF
  At the highest point, lift your forearms off the wall without any lumbar movement.

SHOULDER RANGE OF MOTION

Sleeper's stretch

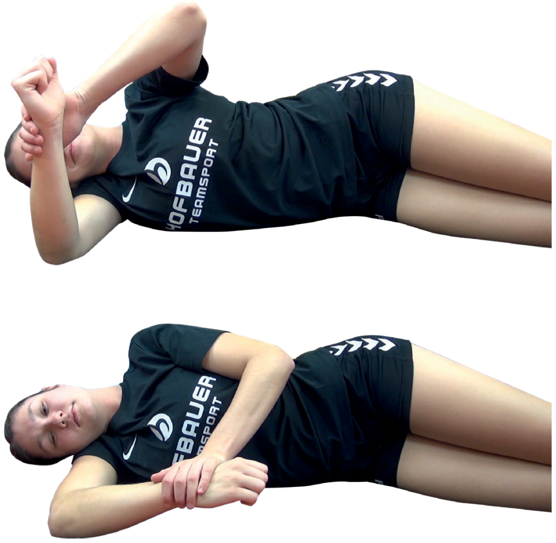

In side-lying position, push your forearm towards the ground and hold.

- <u>ALTERNATIVE EXERCISE: MODIFIED SLEEPER'S STRETCH</u>
  Modify side-lying position by 30° to increase the angle between your trunk and your arm.

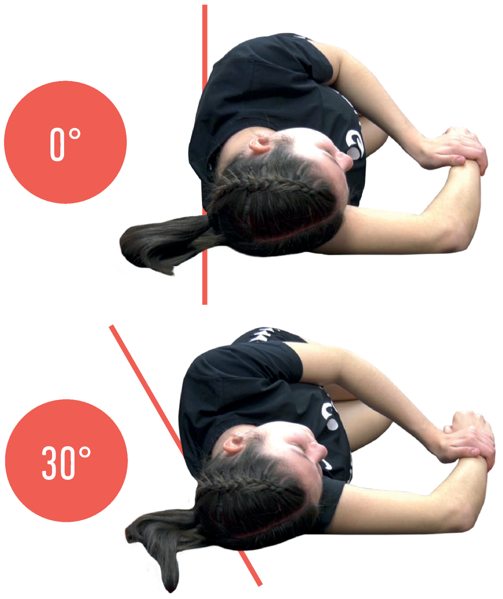

SHOULDER RANGE OF MOTION

Cross-body stretch

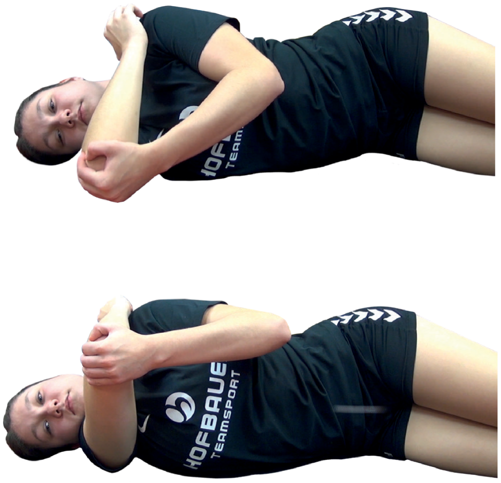

In side-lying position, pull your elbow close to your body and hold.

- ALTERNATIVE EXERCISE: MODIFIED CROSS-BODY STRETCH
  Modify side-lying position by 30° to increase the angle between your trunk and your arm.

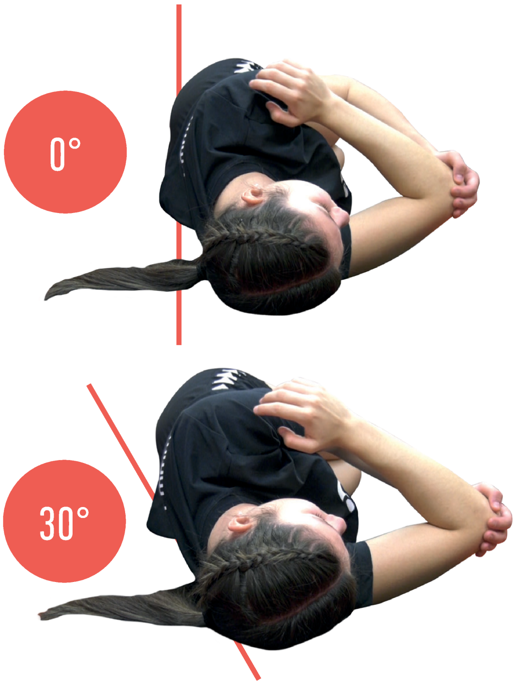

Achenbach L, *et al. BMJ Open Sp Ex Med* 2022; 8:e001270. doi: 10.1136/bmjsem-2021-001270
